# Antiviral Efficacy of the Traditional Chinese Medicine Mixture Yuanzhixingrenheji Against Human Adenovirus-7 In Vitro, In Vivo, and in a Clinical Retrospective Study

**DOI:** 10.3390/pathogens15050463

**Published:** 2026-04-24

**Authors:** Qiuchi Lv, Lexi Li, Ruifei Wang, Shuaibing Han, Hongwei Zhao, Zhengde Xie, Qiang He, Chang Liu, Lili Xu

**Affiliations:** 1 Beijing Key Laboratory of Core Technologies for the Prevention and Treatment of Emerging Infectious Diseases in Children, National Clinical Research Center for Respiratory Diseases, National Key Discipline of Pediatrics (Capital Medical University), Beijing Research Center for Respiratory Infectious Diseases, Beijing Pediatric Research Institute, Beijing Children’s Hospital, Capital Medical University, National Center for Children’s Health, Beijing 100045, China; lvqiuchiped01@126.com (Q.L.); lilexi5607@163.com (L.L.); wrf2091619863@163.com (R.W.); soulflaree@163.com (S.H.); 13361472595@163.com (H.Z.); xiezhengde@bch.com.cn (Z.X.); 2 Research Unit of Critical Infection in Children, Chinese Academy of Medical Sciences, 2019RU016, Beijing 100045, China; 3 Department of Traditional Chinese Medicine, Beijing Children’s Hospital, Capital Medical University, National Center for Children’s Health, Beijing 100045, China; bchhsp@126.com

**Keywords:** human adenovirus, traditional Chinese medicine, antiviral medicine

## Abstract

Human adenovirus type 7 (HAdV-7) is a significant pathogen responsible for viral community-acquired pneumonia in children. To date, no specific antiviral agents have been approved for clinical use against HAdV infections. Traditional Chinese medicine (TCM) mixtures have shown promising potential in managing viral pneumonia. This study aimed to evaluate the antiviral activity of Yuanzhixingrenheji (YZ), a hospital-prepared TCM formulation from Beijing Children’s Hospital, against HAdV-7. Initial screening of four hospital formulations (Feiyanheji, Qingjieheji, Yindaizhikeheji, and Yuanzhixingrenheji) using a CCK-8 assay revealed that YZ exhibited the lowest cytotoxicity. In vitro, YZ pretreatment and post-infection treatment exhibited dose-dependent antiviral activity against HAdV-7 in A549 cells, significantly suppressing the DBP mRNA level and protein expression while reducing viral genome copies, HAdV-7-GFP fluorescence, hexon fluorescence, and DBP nuclear localization. In the hDSG2^+/+^ C57BL/6 mouse model of HAdV-7 infection, YZ effectively mitigated infection-induced body weight loss and substantially reduced viral loads in lung tissue. Furthermore, a clinical retrospective analysis indicated that YZ treatment significantly decreased post-hospitalization serum C-reactive protein levels of pediatric patients with HAdV infection in various disease severities. Compared with conventional treatment, YZ treatment also significantly reduced peak temperature and shortened the duration of fever in children with HAdV infection, supporting its therapeutic potential. In summary, this study provides the first integrated evidence from in vitro, in vivo, and clinical retrospective investigations, demonstrating that the TCM mixture YZ has significant anti-HAdV-7 activity and clinical efficacy. Characterized by a favorable safety profile and low economic burden, YZ is a promising candidate for the treatment of pediatric adenovirus pneumonia.

## 1. Background

Human adenovirus (HAdV) is a nonenveloped, double-stranded DNA virus classified within the genus *Mastadenovirus* of the family *Adenoviridae*. HAdV has achieved global circulation since its initial identification by Rowe et al. in 1953 and remains a persistent challenge to public health security [1]. Its viral genome is approximately 36 kb in length. Structurally, the virion has an icosahedral symmetrical structure and is composed of 13 structural proteins, including seven capsid proteins and six core proteins. The capsid proteins are further subdivided into major (hexon, penton base, and fiber) and minor (proteins IIIa, VI, VIII, and IX) components. The core proteins, which reside internally alongside the viral genome, include pVII, pV, Mu (pX), pIVa2, terminal protein (TP), and the adenovirus-encoded protease [2,3,4,5].

Currently, HAdV is classified into seven species (A–G) encompassing 116 identified genotypes [6,7]. HAdV can lead to a spectrum of clinical manifestations, including respiratory infections, gastrointestinal diseases, and conjunctivitis. This is because different HAdV species exhibit distinct tissue tropisms, thereby leading to diverse clinical manifestations. Respiratory tract infections are primarily linked to species B, C, and E, which commonly present with fever, pharyngitis, tonsillitis, cough, and sore throat. Gastrointestinal infections are chiefly associated with species D, E, and G, particularly in pediatric populations [8]. Ocular infections are predominantly caused by species C and D [9,10].

HAdV is a leading pathogen responsible for pediatric lower respiratory tract infections, accounting for approximately 16% of all cases of viral community-acquired pneumonia (VCAP) in China [11]. Compared with the common pathogen HAdV-3 in children with VCAP, HAdV-7 is more likely to result in severe pneumonia, acute respiratory distress syndrome (ARDS), central nervous system disorders, and death. VCAP caused by HAdV-7 in children has become a major public health concern and poses a serious threat to their health [12,13,14]. From a global perspective, HAdV-7 is the predominant adenovirus type causing severe illness and death in children [15]. Currently, no antiviral drugs that are both effective and safe for the treatment of HAdV infection have been clinically approved. Therefore, the research and development of anti-adenovirus agents is urgently needed [16,17].

During the COVID-19 pandemic, the application of traditional Chinese medicine (TCM) mixtures for viral pneumonia attracted considerable attention. Several TCM mixtures have demonstrated notable clinical therapeutic benefits alongside well-characterized antiviral properties [18,19,20]. Previous studies have indicated that classical Chinese herbal formulas exhibit antiviral or anti-inflammatory effects against adenoviruses. For example, the Chinese herbal formula Maxing Shigan Tang can suppress the expression of the inflammatory mediators, transforming growth factor-β1 (TGF-β1), platelet-derived growth factor-BB (PDGF-BB), and tumor necrosis factor-α (TNF-α) in human embryo lung fibroblasts (HELF) following HAdV-3 infection [21]. Qingreheji exhibits anti-HAdV-3 activity in Vero and Hep-2 cells, whereas Shuanghuanglian tablets demonstrate efficacy in HAdV-3-infected ICR mice in vivo [22,23]. In addition, small-molecule compounds derived from traditional Chinese medicine, such as cardamomin, nuciferine, and *Radix lithospermi*, have been demonstrated to possess well-defined anti-adenovirus activity [16]. However, the previous studies have limitations, as the active ingredients of most of the Chinese herbal formulas mentioned before remain unidentified, and their specific anti-adenoviral mechanisms are not yet fully understood.

In our study, we selected hospital-prepared TCM mixtures from Beijing Children’s Hospital that are routinely used to alleviate pediatric respiratory symptoms resulting from VCAP, such as cough, excessive phlegm, and throat discomfort. Their compositions are listed in the table below (Table 1). Among them, the main components of Feiyanheji (FYHJ) and Qingjieheji (QJHJ)—*Flos Lonicerae* (honeysuckle) and *Forsythia suspensa*—are common constituents of classic TCM formulas known for their heat-clearing and detoxifying effects [24]. Notably, *Forsythia suspensa* has been extensively studied for its broad-spectrum activity against respiratory viruses, including RSV, SARS-CoV-2, and HCoV-229 [25,26]. While *Asiatic Moonseed Rhizome*, a constituent of Yindaizhikeheji (YDZK), has been reported to exert antiviral activities via the modulation of host immune responses [27]. And YZ contains *Ammoniated Anise Spirit* (*Illicium verum* Hook.f.), whose bioactive constituent (−)-bornyl p-coumarate exhibits potent antiviral activity against H1N1, suggesting potential broad-spectrum efficacy against respiratory viruses [28]. Building upon these considerations, we first evaluated the cytotoxicity and preliminary antiviral activity of the four TCM mixtures. The mixture that exhibited the lowest cytotoxicity (YZ) was subsequently chosen for detailed antiviral investigation. An HAdV-7 infection model was established in hDSG2^+/+^ C57BL/6 mice to evaluate the in vivo antiviral efficacy of YZ. Finally, a clinical retrospective study was conducted to validate its therapeutic efficacy in pediatric patients with adenoviral pneumonia. This investigation revealed that YZ exhibits inhibitory effects against HAdV-7.

## 2. Methods

### 2.1. Clinical Specimens and Data Collection

Our study retrospectively analyzed patients with viral pneumonia who were admitted to Beijing Children’s Hospital, Capital Medical University, from 1 January 2017 to 30 June 2025. A total of 1311 cases were initially reviewed. Each case was diagnosed according to the evidence-based guidelines regarding the diagnosis of pneumonia in children published by the World Health Organization [29]. In the clinical efficacy assessment, propensity score matching (PSM) was applied to the baseline covariates of both groups to control for bias, using a matching ratio of 1:2 and a caliper width of 0.02.

Inclusion criteria: (A) HAdV was detected by quantitative real-time polymerase chain reaction (qPCR), with a cycle threshold (Ct) value of less than 40 considered positive; (B) for the YZ treatment group, they were under YZ treatment during the disease course, and for conventional treatment group, they were under treatment with only conventional treatment without any TCM; (C) age of less than 18 years.

Exclusion criteria: (A) Patient had hospital-acquired adenovirus pneumonia; (B) co-infection with other pathogens; (C) incomplete clinical data; (D) patients with immunocompromised status or underlying chronic diseases that could potentially confound the diagnosis.

### 2.2. Cell Lines and Viruses

Human non-small cell lung cancer (A549) cells (Chinese Academy of Medical Sciences) were cultured in Dulbecco’s modified Eagle’s medium (DMEM; Gibco, CA, USA) supplemented with 10% fetal bovine serum (FBS; Gibco, CA, USA) and incubated at 37 °C with 5% CO_2_. HAdV-B7 was stored in our laboratory. HAdV-B7 was passaged in A549 cells cultured in DMEM supplemented with 2% FBS. HAdV-7-GFP was constructed in our laboratory [30]. Viral titers were calculated according to the methods of Kaerber and expressed as the 50% tissue culture infectious dose (TCID_50_) per milliliter.

### 2.3. Antibodies and Chemicals

A rabbit anti-GAPDH antibody (5174S) was purchased from Cell Signaling Technology (Danvers, MA, USA), and a rabbit anti-DBP antibody was prepared in our laboratory. Mouse anti-HAdV-7 hexon antibody (M0018B) for immunofluorescence analysis was purchased from RIDABIO (Guangzhou, China). Horseradish peroxidase-labeled goat anti-rabbit (A0208) and anti-mouse (A0216) secondary antibodies were purchased from Beyotime (Shanghai, China). The four TCMs used in this study—Feiyanheji (FYHJ), Qingjieheji (QJHJ), Yindaizhikeheji (YDZK) and Yuanzhixingrenheji (YZ)—are hospital preparations of Beijing Children’s Hospital, Capital Medical University. Brincidofovir (BCV) was purchased from MedChemExpress (Monmouth Junction, NJ, USA).

### 2.4. Animal Experiments

For the animal experiments, hDSG2^+/+^ C57BL/6 mice were kindly gifted from Professor Liqiang Feng (Guangzhou Institutes of Biomedicine and Health, Chinese Academy of Sciences) and Dr. Ying Feng (Guangzhou National Laboratory) and have been described elsewhere [12]. Eight 4- to 6-week-old hDSG2^+/+^ mice were randomly assigned to a virus control group (HAdV-7/vehicle) or YZ treatment group (HAdV-7/YZ), with four mice in each group. Mice were intranasally inoculated with the HAdV-7 strain at a dosage of 50 μL 1 × 10^10^ TCID_50_ per mouse. At 2 h post virus challenge, mice in the YZ group were administered 200 µL of YZ daily (the dose was converted from the clinical dosage for 7-year-old children), while those in the virus control group received an equal volume of PBS via daily gavage. Following infection, the body weights of the mice and signs of disease were observed on a daily basis, and the mice were humanely euthanized three days post-infection. At the time of death, different tissues were harvested and stored at −80 °C for further analysis.

### 2.5. Cell Titer-Glo Assay for the Cytotoxicity and Antiviral Activity of BCV

The cytotoxicity and antiviral activity of BCV against HAdV were determined using a Cell Titer-Glo Luminescent Cell Viability Assay Kit (Promega, Madison, WI, USA). The Cell Titer-Glo Reagent induces cell lysis and the generation of luminescence proportional to the amount of ATP present in cells. The resulting luminescence intensity was measured using a luminometer (Molecular Devices, San Jose, CA, USA) according to the manufacturer’s instructions. Briefly, A549 cells (10,000 cells/well in 50 µL) were seeded into a white 96-well plate and cultured for 24 h at 37 °C under 5% CO_2_. Subsequently, 5 µL of serially diluted drugs and 45 µL of 2% DMEM/HAdV-7 were added to each well. After 5 days of incubation, 50 µL of Cell Titer-Glo reagent was added to each well according to the manufacturer’s instructions. Following thorough mixing and a 30 min equilibration period, cellular viability was measured using a microplate reader. The cytotoxicity of BCV was determined by comparison with that of a control group treated with only 0.5% DMSO.

### 2.6. CCK-8 Assay of the Cytotoxicity of TCM Mixtures

Cell cytotoxicity of TCM mixtures was determined using a CCK-8 assay kit (Beyotime) according to the manufacturer’s instructions. A549 cells were seeded into 96-well plates at a density of 5000 cells per well in 50 µL 2% DMEM and cultured for 24 h at 37 °C under 5% CO_2_. Then, 50 µL of serially diluted drugs was added to each well. Following incubation for 24 h, the original culture medium was removed, and cells were washed twice with PBS. Next, 100 µL of 2% DMEM containing 10% CCK-8 reagent was added to each well. After incubation in the dark for 30 min, cellular viability was detected using a microplate reader.

### 2.7. Western Blot Analysis

Samples were lysed in radioimmunoprecipitation assay (RIPA) buffer (Beyotime) supplemented with protease inhibitor cocktail (Beyotime) and phosphatase inhibitor cocktail (Beyotime). The cell lysates were centrifuged at 12,000 rpm for 10 min at 4 °C to clear the debris. The protein concentration was determined by a bicinchoninic acid (BCA) assay (Thermo Fisher, Waltham, MA, USA). SDS loading buffer was added to the collected supernatant before heating at 101 °C for 8 min to denature the proteins. The proteins were separated by 10% Tris-glycine gel electrophoresis (SDS–PAGE) before being transferred onto a polyvinylidene difluoride membrane (Roche, Basel, Switzerland). The membranes were blocked for 2 h at room temperature on a shaker with 5% milk. Furthermore, the membranes were incubated with a primary antibody overnight at 4 °C, followed by a second incubation at room temperature for 1–2 h with appropriate HRP-conjugated secondary antibodies. After further washing with PBST, the blots on the membranes were visualized with ECL reagent (Beyotime) according to the manufacturer’s protocol. Equal protein loading was confirmed in all the experiments by using GAPDH as a loading control.

### 2.8. RNA/DNA Extraction and Real-Time Fluorescence Quantitative PCR

Total cell RNA was purified using TRIzol Reagent (Invitrogen, Carlsbad, CA, USA), followed by reverse transcription into cDNA using First-Strand cDNA Synthesis Mix (LABLEAD, Beijing, China) according to the manufacturer’s instructions. Cell culture supernatants were collected after centrifugation, and DNA was extracted using a QIAamp MinElute Virus Spin Kit (QIAGEN, Hilden, Germany) according to the manufacturer’s instructions. The relative gene expression levels and viral genome copies were analyzed using SYBR Green dye (RR067A; TAKARA, China) and specific primer pairs on a real-time fluorescence quantitative PCR instrument (Thermo Scientific, Waltham, MA, USA). The expression levels of the host or viral mRNA were standardized relative to those of the housekeeping gene GAPDH. The following primers were used for quantitative PCR analyses: HAdV-7 DBP forward: 5′-ATGGAAGTGATGGCTGTGCTAATGG-3′; HAdV-7 DBP reverse: 5′-CTTGTACTGCTCGTGCTGCTCTG-3′; HAdV-7 hexon forward: 5′-GGCCAAGCATCACAACTGAA-3′; HAdV-7 hexon reverse: 5′-TCCACAGCCTGATTCCACAT-3′; GAPDH forward: 5′-GATTCCACCCATGGCAAATC-3′; and GAPDH reverse: 5′-CTGGAAGATGGTGATGGGATT-3′.

Total DNA from mouse plasma and lungs was extracted using a QIAamp MinElute Virus Spin Kit (QIAGEN, Hilden, Germany). A series of serial dilutions of the recombinant HAdV-7 DBP plasmid containing the target DNA fragment (or the linearized standard DNA) was prepared to generate a standard curve. The specific steps and primers for real-time fluorescence quantitative PCR were the same as those described above. The copy number of target DNA in the sample was calculated on the basis of the standard curve established by the standard dilutions.

### 2.9. Immunofluorescence Analysis

The cells were fixed with 4% paraformaldehyde (Beyotime, P0099) for 30 min at 26 °C and then blocked with an immunostaining blocking solution (Beyotime, P0260) at 26 °C for 20 min. Subsequently, the corresponding primary antibodies were diluted in diluent buffer at the indicated concentration and incubated with the cells overnight at 4 °C. After washing with TBST, the cells were incubated with fluorochrome-conjugated secondary antibodies (Alexa Fluor 488, 1:500; Cell Signaling Technology, 4408S, 4412S) at 26 °C for 2 h. The cells were then incubated in an antifade mounting medium with DAPI (Beyotime, P0131; Invitrogen, P36966) for 1 h. Fluorescence images were acquired with a fluorescence microscope (Bio-Rad, Zoe Fluorescent Cell Imager, Hercules, CA, USA) or a confocal laser scanning microscope (Leica, SP8, Wetzlar, Germany). The quantitative analysis of colocalized immunofluorescence intensity was performed using ImageJ software (version 1.54f), and approximately 25–30 cells in total for each condition were used for quantification. The images shown are representative of at least three independent experiments.

### 2.10. Statistical Analysis

Data analysis was performed using SPSS (version 22.0) and GraphPad Prism (version 10.0). To ensure the reliability of the experimental results, all the experiments were conducted with three independent biological replicates. A *p*-value of less than 0.05 was considered to indicate statistical significance. The significance levels are denoted as follows: * *p* < 0.05, ** *p* < 0.01, *** *p* < 0.001, and **** *p* < 0.0001.

## 3. Results

### 3.1. Cytotoxicity of the Four TCMs Based on CCK-8

**Figure 1:**
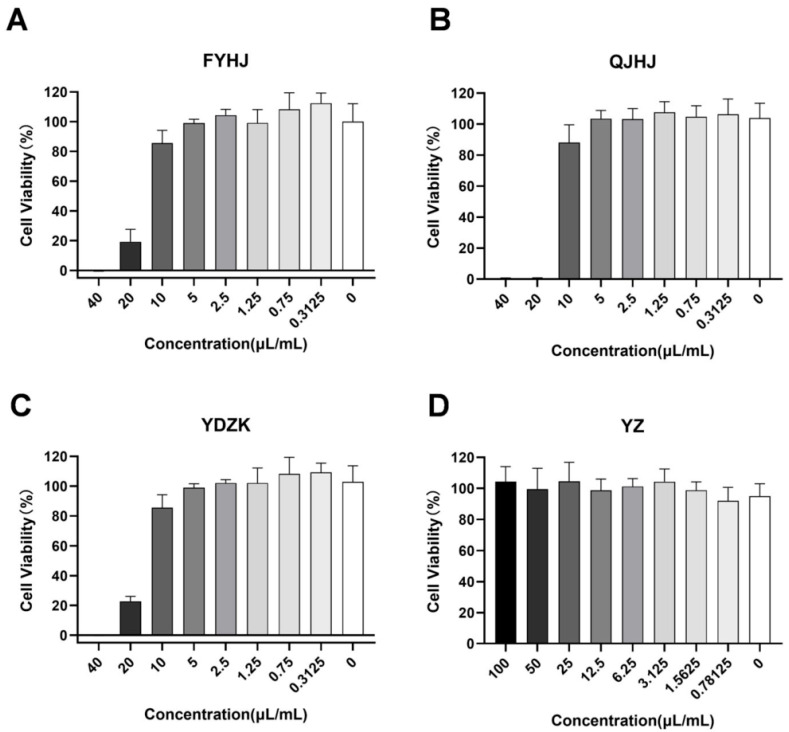
Cytotoxicity of four TCM mixtures. (**A**) FYHJ, (**B**) QJHJ, and (**C**) YDZK were 2-fold serially diluted from 40 µL/mL, and (**D**) YZ from 100 µL/mL, each generating eight concentrations. A549 cells were incubated with the indicated concentrations for 24 h, and the cytotoxicity of the four TCM mixtures on A549 cells was assessed using the CCK-8 assay.

The cytotoxicity of four TCMs commonly used for treating viral pneumonia at Beijing Children’s Hospital (FYHJ, QJHJ, YDZK, and YZ) was assessed using CCK-8. A549 cells were co-incubated with serially diluted drugs for 24 h. Compared with the control group, FYHJ, QJHJ, and YDZK showed no significant cytotoxicity at 10 μL/mL, while YZ exhibited no significant cytotoxicity at 100 μL/mL (Figure 1). Based on these findings, appropriate non-toxic concentrations were selected for the preliminary evaluation of anti-HAdV-7 activity (FYHJ at 6.25 µL/mL, QJHJ at 6.25 µL/mL, YDZK at 10 µL/mL, and YZ at 50 µL/mL).

### 3.2. Inhibitory Effect of YZ on HAdV-7

**Figure 2:**
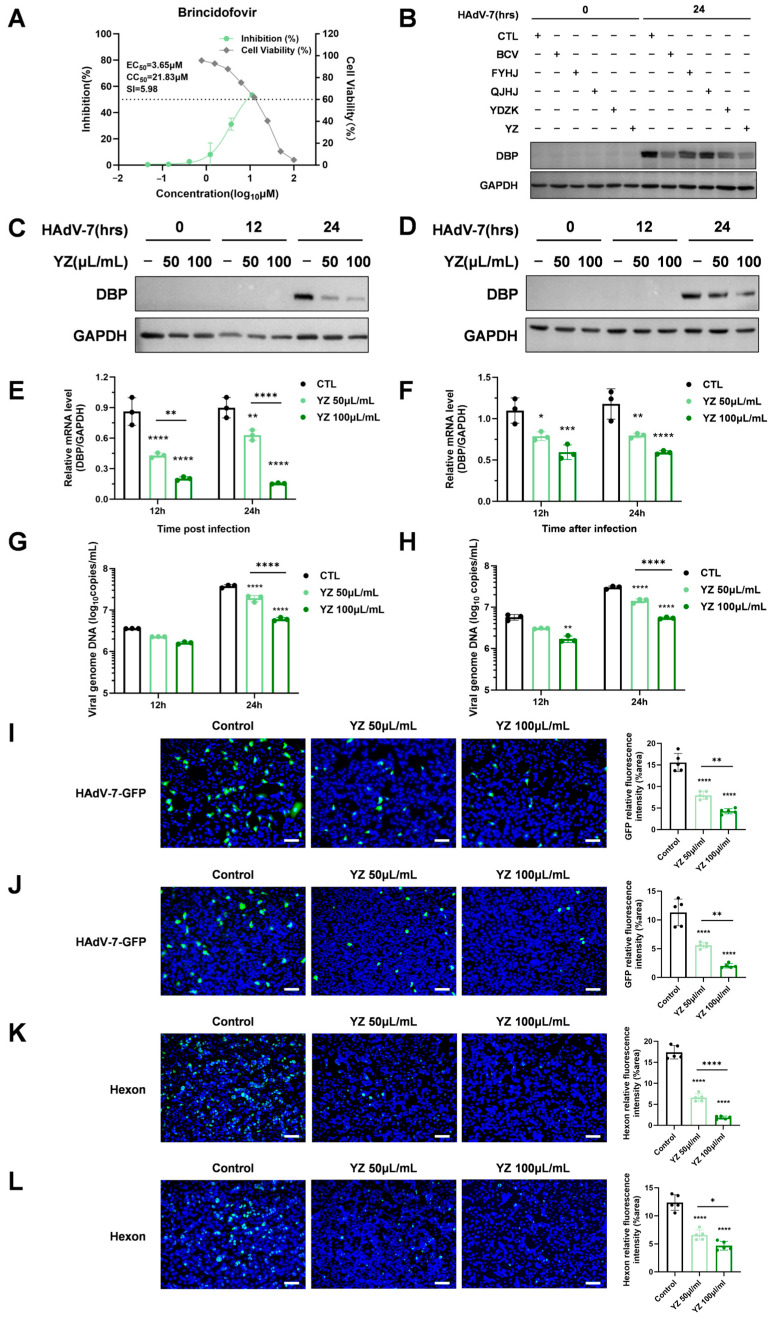
Inhibitory effect of YZ on HAdV-7 in vitro. (**A**) Cell Titer-Glo assay of the cytotoxicity and antiviral activity of BCV. (**B**) Preliminary verification of the antiviral activity of the four TCMs. A549 cells were pretreated with indicated TCMs (FYHJ at 6.25 µL/mL, QJHJ at 6.25 µL/mL, YDZK at 10 µL/mL, and YZ at 50 µL/mL) and 5 μM BCV as a positive control and then infected with HAdV-7 at MOI = 1. The cell lysates were collected at 24 hpi to evaluate the expression of HAdV-7 viral protein DBP by Western blot. (**C**–**H**) A549 cells were treated with YZ (50 µL/mL or 100 µL/mL) 1 h before or after HAdV-7 infection at an MOI of 1. Samples were collected at 12 and 24 hpi for analysis. Western blot analysis of viral protein DBP in the pretreatment group (**C**) and post-infection treatment group (**D**). Relative mRNA expression levels of DBP determined by real-time PCR in the pretreatment group (**E**) and post-infection treatment group (**F**). After pretreatment (**G**) or post-infection treatment (**H**) of YZ, cell culture supernatants were collected for DNA extraction, and viral DNA copies were quantified by qPCR. (**I**–**J**) Immunofluorescence analysis of HAdV-7-GFP infection in A549 cells at 24 hpi under pretreatment (**I**) and post-infection treatment (**J**) conditions. GFP expression (green) was observed via fluorescence microscopy, with DAPI (blue) used to counterstain the nuclei. Relative fluorescence intensity of GFP was quantified using ImageJ software. Scale bar: 100 μm. (**K**,**L**) A549 cells were treated as described above and infected with HAdV-7 under pretreatment (**K**) and post-infection treatment (**L**) conditions. Cells were immunostained with an anti-hexon antibody (green) and DAPI (blue). Relative fluorescence intensity of hexon protein was visualized by fluorescence microscopy and quantified via Image J software. Scale bar: 100 μm. (* indicates *p* < 0.05, ** indicates *p* < 0.01, *** indicates *p* < 0.001, **** indicates *p* < 0.0001).

BCV is a drug with previously verified anti-adenovirus activity [13,14]. As shown in Figure 2A, BCV inhibited the HAdV-7 in a dose-responsive manner with a half-maximal effective concentration (EC_50_) of 3.65 μM and a half-maximal cytotoxic concentration (CC_50_) of 21.83 μM, yielding a selectivity index (SI) of 7.93. Based on these results, BCV was selected as the positive control for subsequent evaluation of the anti-adenovirus effects of TCM mixtures.

In the preliminary screening, A549 cells were pretreated with the five different drugs, infected with HAdV-7 at a multiplicity of infection (MOI) of 1, and assessed at 24 h post-infection (hpi). The viral DNA-binding protein (DBP) encoded by the E2 gene was used to indicate viral infection. Western blot analyses demonstrated that compared with the control treatment, the 5 μM BCV treatment significantly suppressed the expression of the viral DBP. Both YDZK and YZ exhibited comparable anti-HAdV-7 activity (Figure 2B). Consequently, YZ was selected for subsequent antiviral efficacy validation because of its favorable cytotoxicity profile.

Based on the results of the preliminary screening, two concentrations of YZ (50 µL/mL and 100 µL/mL) were selected for further antiviral evaluation. Following pretreatment with YZ, A549 cells were infected with HAdV-7 at MOI of 1, with assessments conducted at 12 and 24 hpi. Western blot analyses and real-time PCR revealed that YZ treatment significantly reduced both the protein and mRNA expression levels of viral proteins DBP (Figure 2C,E). Similar trends were observed at two other MOIs (0.5 and 0.1) (Figure S1A–D). Notably, at an MOI of 1, the mRNA expression of DBP was inhibited in a dose-dependent manner at both time points, suggesting that YZ possesses anti-adenoviral activity. To simulate the process of natural infection followed by therapeutic intervention, A549 cells were treated with YZ at 1 hpi with HAdV-7 at MOI of 1, followed by detection at the same time points. The results demonstrated that post-infection treatment significantly reduced the expression of DBP (Figure 2D,F). But its inhibitory effect on DBP expression was less pronounced compared to that observed under the pretreatment of YZ. To evaluate the effect of YZ on HAdV-7 replication, viral genome DNA copies in cell culture supernatants at 12 hpi and 24 hpi were quantified by qPCR. The results showed that YZ pretreatment and YZ post-infection treatment led to a dose-dependent and significant reduction compared with the control group at 24 hpi (Figure 2G,H). Also, the potent anti-adenoviral activity of YZ was confirmed by immunofluorescence staining. At 24 hpi, the fluorescence intensity of HAdV-7-GFP decreased in a dose-dependent manner compared with the control group in both the YZ pretreatment and post-infection treatment (Figure 2I,J). A similar inhibitory trend was observed for the viral capsid protein hexon following HAdV-7 infection (Figure 2K,L). Furthermore, since DBP typically localizes to the nucleus of infected cells and becomes detectable between 4 and 12 hpi, we examined its nuclear localization at both 12 and 24 hpi [15]. As shown in Figure S1E, DBP expression remained low at 12 hpi, with no significant difference observed between the control and YZ-treated groups. At 24 hpi, DBP expression increased markedly, and YZ treatment led to a dose-dependent reduction in the nuclear accumulation of DBP.

### 3.3. Antiviral Activity of YZ Against HAdV-7 In Vivo

**Figure 3:**
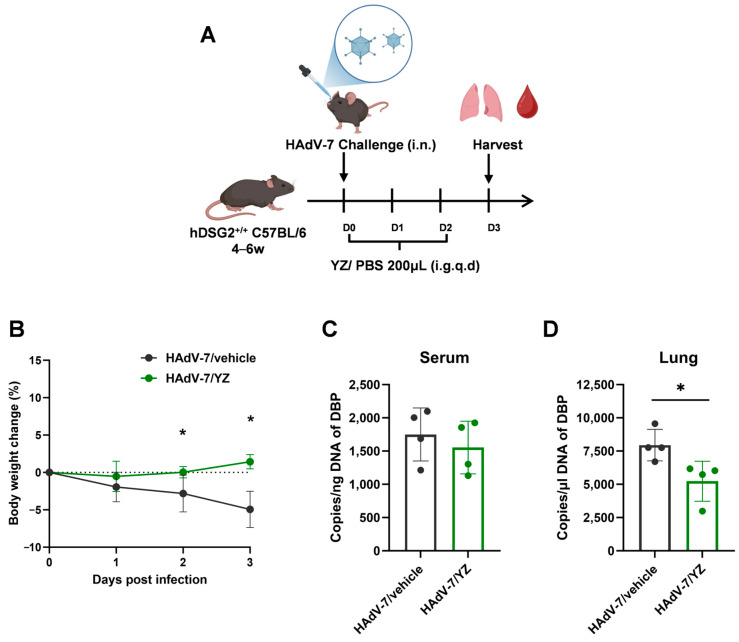
Antiviral activity of YZ against HAdV-7 in vivo. (**A**) Experimental scheme of HAdV-7-infected hDSG2^+/+^ C57BL/6 mouse model. Mice were randomly assigned to the control group (HAdV-7/vehicle, n = 4) or YZ treatment group (HAdV-7/YZ, n = 4). Mice were intranasally inoculated with the HAdV-7 strain at a dosage of 50 μL 1 × 10^10^ TCID_50_ per mouse. At 2 h post virus challenge, mice in the YZ group were administered 200 µL of YZ daily (the dose was converted from the clinical dosage for 7-year-old children), while those in the control group received an equal volume of PBS via daily gavage. Following infection, the body weights of the mice and signs of disease were observed on a daily basis, and the mice were humanely euthanized three days post-infection. (**B**) Body weight was monitored at the indicated time points. Viral loading in (**C**) plasma and (**D**) lung tissue (* indicates *p* < 0.05).

To further evaluate the in vivo antiviral efficacy of YZ, we established a HAdV-7 infection model in hDSG2^+/+^ C57BL/6 mice. Eight 4- to 6-week-old hDSG2^+/+^ mice were randomly assigned to a virus control group (HAdV-7/vehicle) or YZ treatment group (HAdV-7/YZ), with four mice in each group. The mice were intranasally inoculated with the HAdV-7 strain at a dosage of 50 μL 1 × 10^10^ TCID_50_ per mouse. Two hours after virus challenge, YZ group mice received daily administration of 200 μL YZ (dose converted from clinical dosage for 7-year-old children), with virus control mice given an equal volume of PBS by daily gavage (Figure 3A). Following infection, the body weights of the mice and signs of disease were observed on a daily basis, and the mice were humanely euthanized at 3 dpi. As shown in the results, treatment with YZ prevented body weight loss (Figure 3B). To determine the effect of YZ on virus replication, we assayed the plasma and lungs of hDSG2^+/+^ C57BL/6 mice sacrificed at 3 dpi to determine the viral burden. We found that the viral load in the lung tissue of the HAdV-7/YZ group was significantly lower than that in the lung tissue of the HAdV-7/vehicle group (*p* < 0.05), and a similar trend was observed in plasma, although the difference did not reach statistical significance (Figure 3C,D).

### 3.4. Clinical Study of the Antiviral Activity of YZ Against HAdV-7

Our study retrospectively analyzed the medical records of 1311 pediatric patients with viral pneumonia admitted to Beijing Children’s Hospital between 1 January 2017 and 30 June 2025. Among them, 489 patients were diagnosed with adenovirus infection. After excluding patients with coinfections by other pathogens, incomplete clinical data, or underlying chronic conditions, a total of 13 patients receiving YZ treatment and 111 patients receiving conventional treatment were enrolled in the study. Given the imbalance between the two groups, propensity score matching (PSM) was applied to the baseline covariates of both groups to control for the bias, using a matching ratio of 1:2 and a caliper width of 0.02. Following PSM, 13 patients in the YZ group and 26 patients in the conventional treatment group were used for analysis of the clinical efficacy of YZ in treating adenovirus pneumonia. The baseline characteristics of the patients matched via PSM are presented in Table 2. No significant differences were observed between the two groups in terms of gender, age, season of admission, disease severity, admission temperature, or length of hospital day.

**Figure 4:**
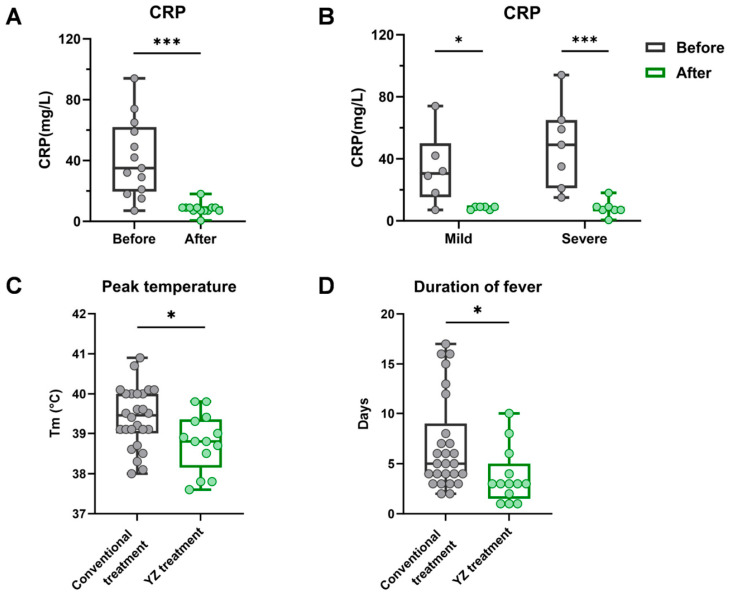
Clinical study of the efficacy of YZ treatment in pediatric patients with HAdV pneumonia. Changes in CRP before and after treatment by YZ (**A**) in all cases and (**B**) in mild/severe cases. “Before” refers to the results of the first CRP test performed immediately upon the patient’s admission, and “After” refers to the results of the final CRP test conducted just prior to the patient’s discharge. Comparison between the YZ group and the conventional group of (**C**) peak temperature and (**D**) duration of fever. (* indicates *p* < 0.05, , *** indicates *p* < 0.001).

C-reactive protein (CRP), an acute-phase reactant, is primarily used in clinical practice to assess the degree of the systemic inflammatory response. Accordingly, we analyzed serum CRP levels in the YZ treatment group before and after treatment. The results showed that treatment with YZ led to a significant reduction in post-treatment CRP levels across all patients, regardless of disease severity (Figure 4A,B). Further analysis of the decrease in serum CRP levels (∆CRP) revealed a greater reduction in the YZ treatment group than in the conventional treatment group, but the difference between the two groups did not reach statistical significance (Table 3). In addition, peak temperature and the duration of fever in the YZ treatment group were significantly lower than those in the conventional treatment group (Figure 4C,D, Table 3). These results indicate that YZ treatment possesses favorable clinical efficacy and can significantly alleviate febrile symptoms in patients.

## 4. Discussion

The identification of effective and safe antiviral interventions for pediatric HAdV-7 infection remains a formidable challenge in global public health [15]. Our study provided the first integrated evidence that the TCM mixture YZ exerts robust antiviral effects through a multi-dimensional approach by demonstrating consistent efficacy across in vitro inhibition, in vivo viral load reduction in hDSG2-transgenic mice, and clinical alleviation of fever. We bridged the gap between in vitro antiviral research and clinical application for TCM. Furthermore, the superior safety profile of YZ relative to traditional agents like Cidofovir (CDV) underscores its potential as a promising candidate for large-scale clinical management of severe pediatric pneumonia.

TCM has been used in China to treat upper and lower respiratory tract infections for thousands of years, and numerous effective TCM prescriptions and drugs have been developed. During the COVID-19 pandemic, the Sanhan Huashi Formula (SHHS) was confirmed to have antiviral effects and was included as a guideline-recommended medication in China, playing a significant role in combating the pandemic [20].

HAdV subtypes predominantly associated with respiratory infections include species B (HAdV-3, 7, 11, 14, 16, 21, 50, and 55), C (HAdV-1, 2, 5, and 6), and E (HAdV-4). Among these serotypes, HAdV-3 and HAdV-7 are the primary serotypes responsible for pediatric adenoviral infections [12,31,32]. The clinical symptoms of adenovirus pneumonia mainly include high fever, cough, wheezing, and lethargy. Severe cases of adenovirus pneumonia are characterized by critical illness, rapid progression, and a prolonged disease course [33]. Clinical treatment is mostly symptomatic. Cidofovir, a cytosine nucleotide analog, inhibits viral DNA polymerase, thereby suppressing viral replication. Studies have shown that early CDV administration may be considered for patients with severe HAdV infection and existing comorbidities [34]. But its low bioavailability and obvious nephrotoxicity have limited its widespread use. The clinical efficacy of CDV in treating HAdV viremia remains a subject of ongoing debate. While a clinical cohort has reported favorable outcomes, including rapid virological response and symptomatic improvement, other studies suggest that CDV lacks definitive therapeutic benefits [34,35]. BCV, a lipid-related derivative of CDV, has good application prospects for the treatment of HAdV infections and is not nephrotoxic [36]. Despite BCV receiving the Fast Track designation for the treatment of HAdV infections, the results from clinical trials were unsatisfactory and showed its toxicity to the gastrointestinal tract [16]. As to the TCM mixture YZ, it has been used clinically for years with a well-established safety profile and defined preparation process. The functions of YZ in treatment are extensive, with the advantages of minimal adverse reactions and low cost. Compared with CDV and BCV, YZ offers a broader therapeutic window and greater safety.

YZ is a hospital-prepared formulation of Beijing Children’s Hospital, primarily aimed at alleviating symptoms such as cough, excessive phlegm, and throat discomfort. In our study, we demonstrated that YZ exhibits antiviral activity against HAdV infection both in vitro and in vivo. Compared with the other three TCM mixtures, YZ had lower cytotoxicity and better antiviral efficacy. These promising characteristics were consistently validated by multiple in vitro experiments demonstrating YZ’s antiviral activity. Furthermore, Ammoniated Anise Spirit, one of the main components of the YZ, is derived from *Illicium verum* Hook.f. (Chinese star anise), a traditional Chinese spice with established antiviral activity. Its bioactive constituent, (−)-bornyl p-coumarate, has been demonstrated to exhibit significant inhibitory effects against the influenza A H1N1 virus [28]. The specific bioactive compounds within YZ responsible for its antiviral effects require further identification and clarification. Natural compounds serve as important sources of lead structures for therapeutic drug development, and elucidating the precise active TCM compounds will facilitate the development of more targeted antiviral agents.

In the in vitro studies, we evaluated two regimens to capture the full spectrum of YZ’s antiviral potential. To maximize the detection of antiviral potential, A549 cells were treated with YZ 1 h before HAdV-7 infection. On the other hand, to simulate the progression of natural infection followed by therapeutic intervention, consistent with our in vivo dosing strategy designed to reflect clinical practice, A549 cells were also treated with YZ at 1 hpi. Our results showed that while post-infection treatment significantly reduced viral genome DNA copies in cell culture supernatants, its inhibitory effect on DBP was less pronounced than that observed in the pretreatment group. Given that DBP is an early-stage protein essential for genome replication, these findings suggest that YZ may exert multi-stage inhibitory effects across the viral life cycle, with a particularly potent impact on the late replication stages [37]. Unified administration protocols will be adopted to further delineate its stage(s) of action once the active components of YZ are identified.

Given the urgent need to study the pathogenicity of HAdV and to evaluate antiviral drugs or vaccines, numerous researchers have attempted to establish animal models for HAdV infection [38]. HAdV-7, the most virulent genotype causing severe respiratory infections in children in China, utilizes CD46 and/or DSG2 as primary receptors, the same as HAdV-55 and other species B HAdV. Rodent cells and mice cannot be productively infected by species B HAdV due to host range restriction factors (HRRF). Recently, Feng et al. generated a transgenic mouse line expressing hDSG2 to establish an animal model with HAdV-55 infection [39]. Zhou et al. also investigated that the animal model of hDSG2 transgenic mice lung infection, established by using nasal instillation of HAdV-55, supports HAdV-55 infection and replication in the lung [40]. The hDSG2+/+ C57BL/6 mouse model employed in this study was susceptible to HAdV-7 infection, and treatment with YZ effectively reduced the viral load in lung tissue and alleviated weight loss. Of note, this model was generated by inserting the hDSG2 gene via CRISPR-Cas9 technology. However, the expression level, tissue distribution, and regulatory mechanisms may differ from those in humans. Moreover, other host factors remain of murine origin, and their interactions with the virus may not fully recapitulate those in humans. Accordingly, our results showed that HAdV-7 replication in this model was modest, insufficient to recapitulate severe infection. Similarly, other exploratory models for adenovirus infection, such as lung organoids, tree shrews, and other humanized mice of adenovirus receptors, each have their own limitations [39,41,42,43]. Therefore, the development of superior adenovirus models is needed to facilitate more effective drug evaluation.

CRP serves as a key biomarker for assessing the degree of systemic inflammatory response. CRP levels are typically elevated during adenovirus infection, and this increase may be more pronounced in patients with severe infection [44,45]. In the clinical retrospective analysis, CRP levels in patients significantly and consistently decreased after YZ treatment, regardless of sex or disease severity, indicating a favorable therapeutic effect. These observations align with the findings from both in vitro and animal experiments, providing robust evidence supporting the antiviral efficacy of YZ. As high fever is a hallmark of adenovirus pneumonia and one of the most concerning clinical indicators for both parents and clinicians, the reduced fever duration and peak temperature in the YZ treatment group hold clear clinical significance [46]. The antipyretic effect of YZ may be attributed to its antiviral and anti-inflammatory properties. Taken together, these results confirm the clinical efficacy of YZ in the treatment of HAdV pneumonia in children.

Our study has certain limitations. Owing to their complex composition, TCM formulations inherently possess multitarget characteristics. YZ may also exhibit analogous antiviral effects against other respiratory viruses. Clarifying the antiviral spectrum of such drugs will better guide clinical application and hold significant importance for the future development of broad-spectrum antiviral research. Additionally, due to the retrospective design of this study, serotype-specific data were not available for the enrolled patients. Therefore, we were unable to assess potential correlations between the clinical efficacy of YZ treatment and specific HAdV serotypes. In future studies, following the identification of the active components of YZ, further studies will be performed to systematically evaluate its antiviral spectrum.

In conclusion, we demonstrated through in vivo and in vitro experiments that YZ can inhibit HAdV-7. A retrospective clinical analysis further supported its clinical efficacy. As a safe, low-cost, and already clinically available hospital-prepared TCM mixture, our findings offer valuable insights into the medicinal value of YZ and its potential for new drug development, contributing to guiding the clinical treatment for pediatric HAdV pneumonia.

## Figures and Tables

**Table 1:** Components of the traditional Chinese medicines.

TCM	Components
Feiyanheji	*Gypsum Fibrosum*, *Herba Ephedrae*, *Flos Lonicerae*, *Forsythia Suspensa*, *Puerariae Lobatae Radix*, *Curcumae Radix*, *Semen Armeniacae Amarae*, *Indigo Naturalis*, *Meretricis Concha*, *Fructus Perillae*, *Licorice Root*
Qingjieheji	*Gypsum Fibrosum*, *Flos Lonicerae*, *Radix Rehmanniae*, *Indigo Naturalis*, *Cortex Lycii*, *Radix Cynanchi Atrati*, *Radix Isatidis*, *Forsythia Suspensa*, *Flos Chrysanthemi*
Yindaizhike	*Indigo Naturalis*, *Semen Ginkgo*, *Semen Ginkgo*, *Pinelliae Rhizoma Praeparatum*, *Raphani Semen* (stir-fried), *Concretio Silicea Bambusae*, *Radix Angelicae Dahuricae*, *Asiatic Moonseed Rhizome*, *Cortex Lycii*, *Perilla Stem*
Yuanzhixingrenheji	*Polygalae Tincture*, *Ammoniated Anise Spirit*, *Benzaldehyde Cyanohydrin Solution*

**Table 2:** Comparison of baseline covariates between conventional treatment group and YZ treatment groups.

	Conventional Treatment (n = 26)	YZ Treatment (n = 13)	*p*-Value
Gender			
Male	13	6	0.821
Female	13	7	
Age [M (Q1, Q3)]	4 (2.25, 6)	2 (1, 4)	0.138
Admission season			
Spring	4	1	0.826
Summer	2	2	
Autumn	7	4	
Winter	11	6	
Severity			
Mild	12	6	1
Severe	14	7	
Admission temperature [M (Q1, Q3)]	37.7 (36.7, 38.275)	37.6 (36.6, 37.9)	0.792
Length of hospital stay [M (Q1, Q3)]	11 (9, 15.75)	9 (7, 12)	0.161

**Table 3:** Comparison of the duration of fever and peak temperature after admission between conventional treatment group and YZ treatment group.

	Conventional Treatment (n = 26)	YZ Treatment (n = 13)	*p*-Value
CRP [M (Q1, Q3)]			
Before	28 (22, 47.5)	35 (21, 59)	0.397
After	9 (9, 9)	9 (7, 9)	0.196
△CRP	20 (13.35, 38.4)	26 (20.5, 52)	0.274
Peak temperature [M (Q1, Q3)]	39.45 (39.1, 40)	38.8 (38.5, 39.3)	0.020
Duration of fever [M (Q1, Q3)]	5 (4, 7.75)	3 (2, 4)	0.021

## Data Availability

The datasets supporting the conclusions of this article are included within the article.

## Supplementary Materials

The following supporting information can be downloaded at: https://www.mdpi.com/article/10.3390/pathogens15050463/s1. Figure S1. YZ inhibits adenovirus replication in A549 cells. A549 cells were pretreated with 50 µL/mL or 100 µL/mL of YZ, then infected with HAdV at (A) and (B) MOI = 0.5, (C,D) MOI = 0.1. (E) A549 cells were treated as described above and infected with HAdV-7 for 12 or 24 hpi. Cells were stained with an antibody against DBP (green) and DAPI to label nuclear and were then examined by confocal microscopy. Scale bars: 25 μm. (** indicates *p* < 0.01, *** indicates *p* < 0.001, **** indicates *p* < 0.0001).

## Abbreviation

ARDS	acute respiratory distress syndrome
BCV	Brincidofovir
CC_50_	half-maximal cytotoxic concentration
CDV	Cidofovir
CRP	C-reactive protein
DBP	DNA-binding protein
EC_50_	half-maximal effective concentration
FYHJ	Feiyanheji
HAdV	human adenovirus
HELF	human embryo lung fibroblasts
HRRF	host range restriction factors
LOS	length of hospital stay
MOI	multiplicity of infection
PDGF-BB	platelet-derived growth factor-BB
PFU	plaque-forming units
PSM	propensity score matching
QJHJ	Qingjieheji
RIPA	radioimmunoprecipitation assay
SHHS	Sanhan Huashi Formula
SI	selectivity index
TCM	traditional Chinese medicine
TGF-β1	transformation growth factor-β1
TNF-α	tumor necrosis factor-α
VCAP	viral community-acquired pneumonia
YDZK	Yindaizhikeheji
YZ	Yuanzhixingrenheji
