# Supplementary figures and images for: Antiviral Efficacy of the Traditional Chinese Medicine Mixture Yuanzhixingrenheji Against Human Adenovirus-7 In Vitro, In Vivo, and in a Clinical Retrospective Study
Source: Pathogens. 2026 Apr 24;15(5):463. doi: 10.3390/pathogens15050463 (PMC13209556; doi:10.3390/pathogens15050463)

Figure S1

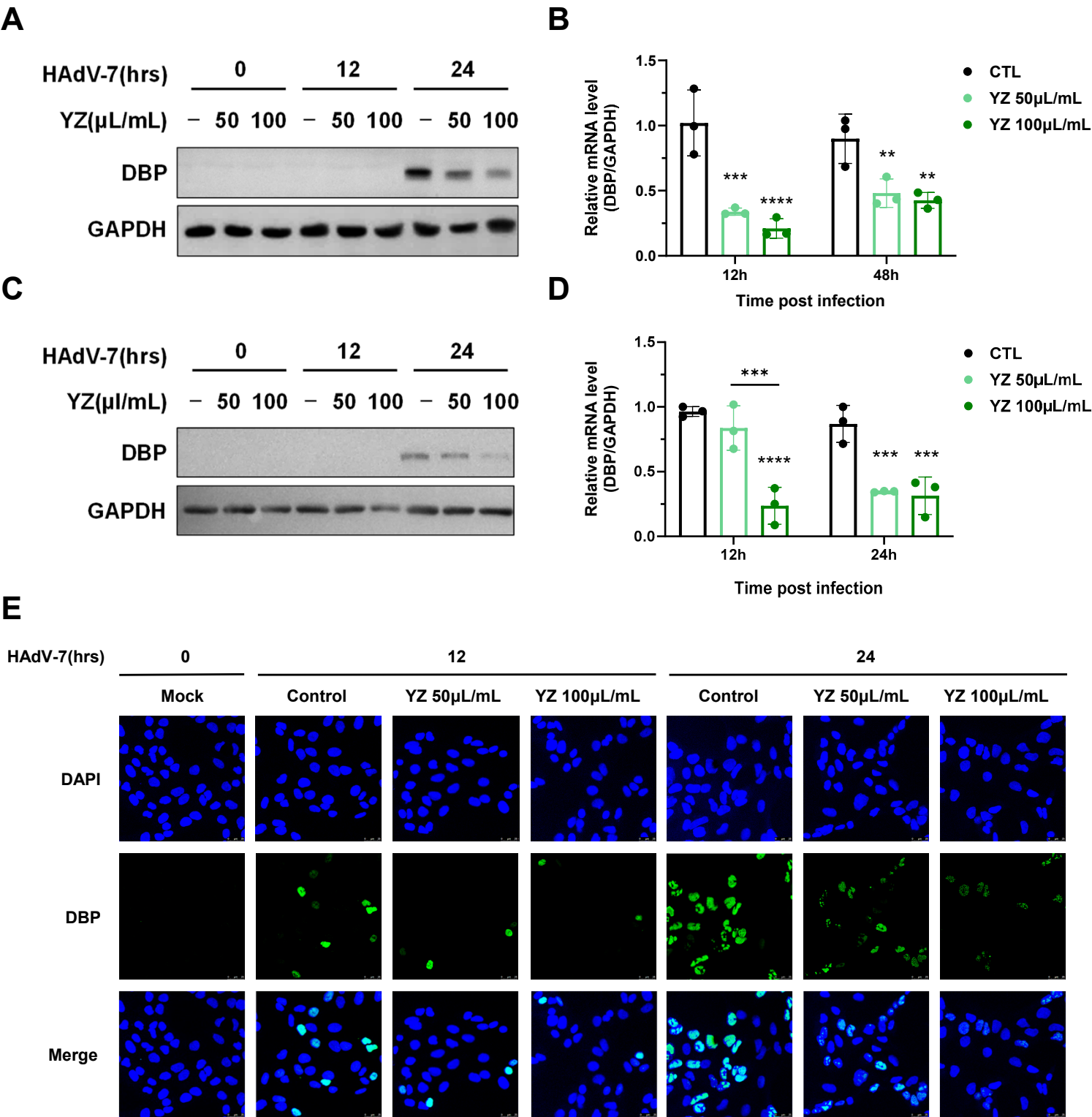

Supplement: Supplementary file 1 [file pathogens-15-00463-s001.zip › pathogens-4229045-supplementary.pdf]
